# Health insurance status and severe mpox disease outcomes among sexual minority men in NYC: a retrospective cohort study

**DOI:** 10.1186/s12916-025-04252-2

**Published:** 2025-07-09

**Authors:** Ofole Mgbako, Cecilia Castellano, Kathryn Jano, Anthony Lo Piccolo, Madeline A. DiLorenzo, Dorothy Knutsen, Yusra Shah, Joyce C. Pressley, Dustin T. Duncan, Jason Felder, Dana Mazo

**Affiliations:** 1 Department of Medicine, NYU Grossman School of Medicine, New York, USA; 2 Department of Population Health, NYU Grossman School of Medicine, New York, USA; 3 Department of Epidemiology, Columbia Mailman School of Public Health, New York, USA; 4 Department of Medicine, BronxCare Health System, New York, USA; 5 NYC Health + Hospitals/Bellevue, New York, USA

**Keywords:** Sociostructural factors, Mpox, Severe disease, Health insurance, Inequities

## Abstract

**Background:**

The 2022–2023 global mpox outbreak predominantly affected sexual minority men, with notable racial/ethnic disparities in the USA. While the current literature has established the clinical predictors of severe mpox disease, little is known about the role of insurance status on clinical outcomes. We sought to characterize patients diagnosed with mpox in New York City (NYC) and examine associations between insurance status and mpox severity score (mpox-SS).

**Methods:**

We included 143 patients aged 18 years and older between May 1, 2022, and December 31, 2023, with confirmed mpox identified through the electronic medical record. Demographics and clinical characteristics were summarized. Linear regression was performed to examine associations between insurance status and mpox-SS, controlling for race/ethnicity, high-risk condition (e.g., HIV with CD4 < 350 cells/mm^3^), prior vaccination with JYNNEOS or a smallpox vaccine, presence of a sexually transmitted infection (STI), and CDC Social Vulnerability Index.

**Results:**

The mean age (SD) was 38.3 (10.2) years with 53 (37.1%) identifying as non-Hispanic White, 44 (30.8%) as Hispanic/Latino, and 30 (20.9%) as non-Hispanic Black. Over 90% were male sex at birth or identified as cisgender men and approximately 80% were sexual minority men. Ninety-six (67.1%) had private insurance, 6 (4.2%) Medicare, 35 (24.5%) Medicaid, and 4 were (2.8%) uninsured. Sixty-three (44.1%) had a confirmed HIV diagnosis, 25 (17.4%) patients had prior JYNNEOS vaccination, and 31 (21.7%) had a high-risk condition. Thirty-eight (26.6%) patients received tecovirimat; 21 (14.7%) patients were hospitalized, with 4 (2.8%) of those admitted to the ICU. The mean (SD) mpox-SS was 6.85 (3.36). In univariate analysis, lack of insurance or Medicaid status was associated significantly with more severe mpox-SS. Insurance status remained significant (*p* = 0.03) in multivariable models.

**Conclusions:**

Being uninsured or on Medicaid was significantly associated with a higher mpox-SS in this diverse cohort of predominantly cisgender sexual minority men in NYC. High-risk status and lack of prior vaccination were associated with higher mpox-SS. Further studies are needed to assess the relationship between insurance, delays in access to care, or other socioeconomic inequities with severe mpox to understand the inequities beyond insurance access to prevent disparities in future outbreaks.

## Background

New York City (NYC) was the epicenter of the mpox outbreak in the summer of 2022, comprising 20% of daily US cases during the peak with around 4000 total cases through December 31, 2022 [1]. Mpox disproportionately impacted sexual minority men (SMM), with 94% of cases occurring among gay and bisexual men and most cases occurring among Hispanic/Latino patients (34.7%), followed by Black/African American patients (27.5%), and non-Hispanic White (22.4%) patients [1]. After the release of the JYNNEOS vaccine for mpox, similar racial inequities in vaccine access compounded the disproportionate burden of disease in Black and Latino gay and bisexual men, who experienced higher hospitalization rates, morbidity, and mortality [1].

The current mpox literature has focused on clinical factors leading to severe mpox outcomes. Uncontrolled HIV and other immunocompromising conditions (e.g., organ transplant, chemotherapy, or conditions such as psoriasis that affect skin integrity) are risk factors for both mpox transmission and severe disease requiring hospitalization [2–8]. Furthermore, most patients who died from mpox in the USA were Black with AIDS-defining illness [9, 10]. Those presenting with or at risk for severe disease were eligible for tecovirimat, an antiviral medication, via a Centers for Disease Control (CDC) expanded access investigational new drug (EA-IND) protocol. In NYC, over 30% of patients diagnosed with mpox received tecovirimat [11]. While a study of roughly 3700 patients with mpox found no association between neighborhood poverty level and tecovirimat prescription, there were treatment disparities by race/ethnicity, with White individuals representing the highest percentage of those treated compared to all other racial groups [11].

Racial/ethnic minority status is a predictor of mpox virus acquisition and infection, poor clinical outcomes, and a lower likelihood of receiving treatment [9, 10, 12]. However, further studies are necessary to understand which socioeconomic factors may contribute to more severe mpox outcomes along the clinical care cascade. Lash et al. found no association between neighborhood poverty level and tecovirimat prescription; however, further studies exploring specific factors such as insurance status are needed [11]. Insurance directly impacts access to healthcare services. Without adequate insurance, individuals may delay or forgo preventive care, diagnostic testing, and treatment due to cost, exacerbating health disparities among underserved populations such as SMM [13–18]. Additionally, lack of insurance often limits access to high-quality providers and specialized care, further increasing the burden of disease across vulnerable populations.

This retrospective cohort study of patients diagnosed with mpox in a NYC academic medical center seeks to address gaps in our knowledge by examining the association between insurance status and mpox severity, controlling for sociodemographic and clinical variables. We hypothesize that lack of insurance or public insurance status is associated with worse outcomes and structural barriers to receiving timely care during this emergent outbreak.

## Methods

### Study setting and design

This retrospective observational cohort study was conducted within a NYC academic medical center, which includes three acute care hospitals and multiple outpatient clinic sites in NYC. We included patients 18 years and older with a confirmed mpox diagnosis in the electronic medical record (EMR) between May 1, 2022, and December 31, 2023, ensuring adequate analysis that captures the height of the outbreak. A confirmed mpox diagnosis was defined by the following inclusion criteria: ICD-10 code for mpox disease; a positive orthopoxvirus PCR result; or a diagnosis in the patient’s problem list, which was manually confirmed via review of clinic notes.

### Data sources and collection procedures

Structured data was first extracted from the EMR, based on the aforementioned inclusion criteria. To address unstructured data and incomplete entries that were not initially extracted from the EMR (e.g., number of lesions, confluent status, or race), patient charts were divided equally among eight authors, who conducted a manual chart review of clinic notes and lab results during mpox infection. A codebook was designed and incorporated for systematic chart review of both extracted data elements and the following incomplete or unstructured data elements: race, psychiatric diagnoses, presence of sexually transmitted infections, highest level of care during mpox illness, reason for hospitalization, reason for tecovirimat prescription, and domains related to mpox severity score. If authors were unsure how to interpret something in a patient chart, they marked it for group discussion. In a group meeting, the authors went through each patient with questions marked for group discussion and came to a collective consensus. The study conforms to the Strengthening the Reporting of Observational Studies in Epidemiology (STROBE) guidelines for observational studies.

### Outcome variables

#### Composite severe mpox disease

Composite severe disease was a binary variable—yes or no. The following were considered markers of severe disease: receipt of tecovirimat to prevent or treat severe disease, hospitalization at any time due to mpox severity, and death. If patients were hospitalized for non-medical reasons other than disease severity, such as no option for safe isolation (i.e., unhoused or residing in congregate settings), they were not included in the composite outcome. Chart review of clinic notes also allowed clarification of tecovirimat receipt specifically due to a severe mpox presentation rather than to prevent disease progression.

#### Mpox severity score (mpox-SS)

The mpox-SS is a 7-item scoring system developed in 2023 that evaluates the following domains: number of active lesions, anatomic extent of lesion involvement, presence of confluent lesions, presence of bacterial superinfection, extent of mucosal areas affected, level of care, and analgesia requirement [19]. The score ranges from 0 to 23. Studies have validated the score as it correlates with the severity of illness in expected groups (e.g., HIV and CD4 count < 200 cells/mm^3^) [20–22]. Clinicians and experienced mpox researchers conducted a manual chart review for the aforementioned factors to calculate a score for each patient from documented encounters in the EMR. For cases with multiple clinical encounters, the encounter closest to 10 days after symptom onset was used.

### Explanatory variables

#### Insurance

The categories were private, Medicare, Medicaid, uninsured, AIDS Drug Assistance Program (ADAP), or unknown [23, 24].

### Sociodemographic covariates

The following variables were collected: age at diagnosis, race, ethnicity, sex at birth, sexual orientation, gender identity, risk factor (e.g., SMM), preferred language, type of health insurance coverage, and zip code of residence.

#### Race/ethnicity

Race/ethnicity was combined into one categorical variable with 4 levels: non-Hispanic Black, non-Hispanic White, Hispanic, and other. If patients were of Hispanic ethnicity, they were coded as Hispanic. This covariate was selected due to multiple studies showing the impact of race on severe outcomes [10].

#### Borough

Zip code was categorized into NYC boroughs (i.e., Manhattan, Brooklyn, Bronx, Queens, and Staten Island) and outside of NYC in NY state. This covariate was selected as an exploratory variable.

#### Social Vulnerability Index (SVI)

Zip code was used to calculate the SVI. The CDC SVI is a score that uses socioeconomic status, household characteristics, racial and ethnic minority status, housing type, and transportation to score counties [25]. We used national SVI, which normalizes each county zip code (organized within census tracts) to a nationwide SVI. SVI was a numeric variable from 0 to 1, from least vulnerable (0) to most vulnerable (1). For example, an SVI score of 0.8 means that 80% of census tracts in the nation are less vulnerable than the tract of interest. This covariate was selected as an exploratory variable.

### Clinical covariates

The following clinical covariates were collected to further characterize the cohort: HIV, prior vaccination, high-risk underlying immunocompromising conditions (e.g., HIV with CD4 count < 350 cells/mm^3^ or acquired immunodeficiency syndrome [AIDS] defining illness), substance use disorder, and mental health disorder [26]. For all clinical covariates, ICD-10 codes were initially used to extract existing data. A subsequent chart review was then completed to discern whether there was information warranting the inclusion of the condition on the patient’s problem list.

#### Prior vaccination

Prior JYNNEOS vaccination was operationalized as a dichotomous variable—yes or no. Prior vaccination was coded as yes if the patient had at least one documented vaccination against orthopoxviruses at least 7 days prior to mpox diagnosis. This covariate was selected due to literature regarding the protective nature of prior vaccination on severe mpox outcomes [27].

#### High-risk condition

High-risk condition was operationalized as a dichotomous variable—yes or no. Conditions were considered high-risk based on the eligibility for tecovirimat described in the EA-IND protocol [28]. High-risk condition was coded as yes if any of the following were present: HIV and either CD4 < 350 cells/mm^3^ OR active AIDS-defining illness including mycobacterium avium complex, cryptococcal disease, Kaposi’s sarcoma, endemic fungal disease; solid organ or hematological transplantation; high dose steroids; skin-compromising condition (i.e., psoriasis, atopic dermatitis, herpes zoster). If none of these conditions was present or if they were unknown after manual chart review, high risk was categorized as no. These covariates were selected because these conditions are understood as risk factors for severe disease [6, 8, 29–32].

#### Mental health disorder

Mental health was operationalized as a dichotomous variable—yes or no. Mental health disorders included an ICD-10 code diagnosis for generalized anxiety disorder, major depressive disorder, bipolar disorder, post-traumatic stress disorder, or schizophrenia. This covariate was selected as an exploratory variable.

#### Substance use disorder

Substance use was operationalized as a dichotomous variable—yes or no. Substance use disorder included an ICD-10 coded diagnosis for marijuana, cocaine, crack, K2, methamphetamine, or alcohol. This covariate was selected as an exploratory variable.

#### Sexually transmitted infection (STI)

STI was operationalized as a dichotomous variable—yes or no. An active STI diagnosis included an ICD-10 coded diagnosis for gonorrhea, chlamydia, syphilis, mycoplasma genitalium, herpes simplex virus, or human papillomavirus 1 month before or after mpox diagnosis. This covariate was selected given association between other STIs and severe mpox disease [33].

Observations with unknown status for some variables are the result of absent documentation in the electronic medical record (EMR).

### Statistical analysis

Eight patients did not have enough clinical information in the EMR to calculate an mpox-SS and were excluded from the regression analysis, leaving 135 patients. Descriptive statistics were used to characterize insurance status, demographic covariates, clinical covariates, and outcomes of the patient population overall [23, 24].

For the regression analysis, the following variables were included: insurance, age, gender identity, sexual orientation, race/ethnicity, language, SVI, STI, prior vaccination, and high-risk status. A dichotomous variable was used with private insurance and/or Medicare versus uninsured or other forms of public insurance (e.g., Medicaid, ADAP). Other epidemiological studies have grouped together lack of insurance and Medicaid insurance given they are both associated with lower income level. Medicare was grouped with private insurance because Medicare eligibility is based on age-requirement rather than income level.

Age was operationalized as a continuous variable. Sex at birth was a categorical variable: male, female, and unknown. Gender identity was a categorical variable: cisgender man, transgender man, cisgender woman, transgender woman, nonbinary/nonconforming, questioning, and unknown. Sexual orientation was a categorical variable: straight, gay, bisexual, lesbian, something else, unsure, decline to answer, and unknown. SMM was a binary variable of yes or no after chart review of clinician notes for risk factors for mpox transmission. Language was a categorical variable: English, Spanish, and other. Race/ethnicity was categorized as above in the Race/ethnicity section. For the regression analysis, we used non-Hispanic White as the reference level and Asian, multiracial/other, and Native American identities were not included given small subgroup sizes. SVI was a continuous variable.

Bivariable associations between the composite mpox-SS and covariates were conducted using chi-squared test for categorical variables and *t*-tests for continuous variables. Covariates were assessed for confounding. Clinical characteristics in Table 2 (excluding substance use and mental health) were considered for inclusion in the multivariable model. Variables significantly associated with severe mpox (*p* < 0.05) were included in the multivariable regression model.

We also examined the correlation between each covariate and the outcome variable. Selected covariates were added to the regression model sequentially, and changes in the *R*-squared value were used to assess improvements in model fit. Covariates known in the literature or hypothesized to be associated with severe mpox, including race and Hispanic ethnicity, were included a priori. The data analysis for this paper was generated using SAS Software Version 14 Online for Academics [34].

### Ethics

This study received approval from the NYU Langone Health Institutional Review Board (IRB), i22-01375.

## Results

The study cohort included 143 patients aged 18 years and older diagnosed with mpox between May 2022 and December 2023 (Table 1). The mean age (SD) was 38.3 (10.2) years and the median (range) was 37 (19–73) years. Fifty-three (37.1%) identified as non-Hispanic White, 44 (30.8%) identified as Hispanic/Latino, 30 (20.9%) identified as non-Hispanic Black, and the remaining patients identified as Asian, Native American, multiracial/other, or unknown. Most (*n* = 124, 91.7%) were male sex at birth and (*n* = 137, 95.8%) identified as cisgender men. Most (*n* = 113, 79%) were SMM. A significant number (*n* = 69, 48.3%) did not have information in the chart regarding sexual orientation, but those providing this information were gay (*n* = 54, 37.7%) followed by as bisexual 9 (*n* = 9, 6.3%) and straight (*n* = 9, 6.3%). Most (*n* = 134, 93.7%) used English as their primary language. The majority (*n* = 96, 67.1%) had private insurance. Those with public insurance had Medicare (*n* = 6, 4.2%), Medicaid (*n* = 35, 24.5%), and ADAP (*n* = 2, 1.4%). Four (2.8%) were uninsured. The majority of patients lived in Manhattan (*n* = 56, 39.2%) and Brooklyn (52, 36.4%) with the remainder in the other three boroughs (*n* = 17, 11.9%) or outside NYC (*n* = 8, 5.6%).

**Table 1:** Demographics by severe disease status, 2022–2023

	Severe disease *N* (%)		
	No (*N* = 98)	Yes (*N* = 45)	All (*N* = 143)
Age, mean (SD)	38.1 (10.2)	38.7 (10.2)	38.3 (10.2)
Race/ethnicity			
Non-Hispanic White	37 (37.8)	16 (35.6)	53 (37.1)
Non-Hispanic Black	22 (22.5)	8 (17.8)	30 (21.0)
Asian	4 (4.1)	2 (4.4)	6 (4.2)
Hispanic/Latino	28 (28.6)	16 (35.6)	44 (30.1)
Native American	2 (2.0)	1 (2.2)	3 (2.1)
Multiracial/other	2 (2.0)	1 (2.2)	3 (2.1)
Unknown	3 (3.06)	1 (2.2)	4 (2.8)
Sexual minority men			
Yes	78 (79.6)	35 (77.8)	113 (79.0)
No	7 (7.1)	4 (8.9)	11 (7.7)
Unknown	13 (13.3)	6 (13.3)	19 (13.3)
Sexual orientation			
Gay	38 (38.8)	16 (35.6)	54 (37.8)
Straight/heterosexual	8 (8.2)	1 (2.2)	9 (6.3)
Bisexual	5 (5.1)	4 (8.9)	9 (6.3)
Declined	0	1 (2.2)	1 (0.7)
Something else	1 (1.0)	0	1 (0.7)
Unknown	46 (46.9)	23 (51.1)	69 (48.3)
Gender identity			
Cisgender man	94 (95.9)	43 (95.6)	137 (95.8)
Cisgender woman	2 (2.0)	1 (2.2)	3 (2.1)
Nonbinary	1 (1.0)	1 (2.2)	2 (1.4)
Transgender man	1 (1.0)	0	1 (0.7)
Language			
English	91 (92.9)	43 (95.6)	134 (93.7)
Spanish	5 (5.1)	2 (4.4)	7 (4.9)
Russian	1 (1.0)	0	1 (0.7)
ASL	1 (1.0)	0	1 (0.7)
Borough			
Manhattan	42 (42.9)	14 (31.1)	56 (39.2)
Queens	4 (4.1)	4 (8.9)	8 (5.6)
Brooklyn	33 (33.7)	19 (42.2)	52 (36.4)
Bronx	5 (5.1)	3 (6.7)	8 (5.6)
Staten Island	1 (1.0)	0	1 (0.7)
NY State	13 (13.3)	5 (11.1)	18 (12.6)
Insurance			
Private	68 (69.4)	28 (62.2)	96 (67.1)
Medicare	4 (4.1)	2 (4.4)	6 (4.2)
Medicaid	22 (22.5)	13 (28.9)	35 (24.5)
Uninsured	3 (3.06)	1 (2.2)	4 (2.8)
ADAP	1 (1.0)	1 (2.2)	2 (1.4)

*ADAP* AIDS Drug Assistance Program, *ASL* American Sign Language

Regarding clinical characteristics, 63 (44.1%) had a confirmed HIV diagnosis (Table 2), of which 42 (67%) had a documented CD4 count in the chart. The median (range) CD4 count was 546.5 (5–1055) cells/mm^3^. Twenty-five (17.4%) patients had a prior mpox vaccination, 31 (21.7%) met the definition of high-risk, and 45 (31.7%) met the criteria for severe disease. Thirty-eight (26.6%) patients received tecovirimat—of those, 37 met severe disease criteria as defined by EA-IND and one met EA-IND criteria for high risk for developing severe disease. Twenty-one (14.7%) patients were hospitalized on the inpatient floor, and four (2.8%) required ICU care. Four (2.8%) patients were for non-medical reasons.

**Table 2:** Clinical characteristics by severe disease status, 2022 to 2023

	Severe disease *N* (%)		
	No (*N* = 98)	Yes (*N* = 45)	All (*N* = 143)
HIV			
Yes	39 (39.8)	24 (53.3)	63 (44.1)
No	59 (60.2)	21 (46.7)	80 (55.9)
Mental health condition^a^			
Yes	39 (39.8)	12 (26.67)	51 (35.7)
No	59 (60.2)	33 (73.3)	92 (64.3)
Substance use^b^			
Yes	26 (26.5)	6 (13.3)	32 (22.4)
No	72 (73.5)	39 (86.7)	111 (77.6)
High risk^c^			
Yes	15 (15.3)	16 (35.6)	31 (21.7)
No	83 (84.67)	29 (64.4)	112 (78.3)
Sexually transmitted infection^d^			
None	73 (74.5)	29 (64.4)	102 (71.3)
1 STI	22 (22.5)	12 (26.7)	34 (23.8)
≥ 2 STIs	3 (3.1)	4 (8.9)	7 (4.9)
Prior vaccination^e^			
Yes	16 (16.3)	9 (20.0)	25 (17.5)
No	82 (83.7)	36 (80.0)	118 (82.5)
Tecovirimat			
Yes	0	38 (84.4)	38 (26.6)
No	98 (100.0)	7 (15.6)	105 (73.4)
Hospital level of care			
Inpatient	2 (2.0)	19 (42.2)	21 (14.7)
Intensive care unit	0	4 (8.9)	4 (2.8)
Outpatient/emergency department	96 (98.0)	22 (48.9)	118 (82.5)

^a^Mental health conditions include documented diagnoses such as depression, anxiety, bipolar disorder, schizophrenia, or other psychiatric disorders

^b^Substance use includes recent or active use of alcohol, tobacco, cannabis, opioids, stimulants, or other recreational drugs

^c^High-risk condition was coded as yes if any of the following were present: HIV and either CD4 < 350 cells/mm^3^ OR active AIDS-defining illness including mycobacterium avium complex, cryptococcal disease, Kaposi’s sarcoma, endemic fungal disease; solid organ or hematological transplantation; high dose steroids; skin-compromising condition. If none of these conditions was present or if they were unknown after manual chart review, high risk was categorized as no

^d^Sexually transmitted infection was operationalized as a dichotomous variable—yes or no. An active STI diagnosis included an ICD-10 coded diagnosis for gonorrhea, chlamydia, syphilis, mycoplasma genitalium, herpes simplex virus, or human papillomavirus 1 month before or after mpox diagnosis

^e^Prior vaccination: prior JYNNEOS vaccination was operationalized as a dichotomous variable—yes or no. Prior vaccination was coded as yes if the patient had at least one documented vaccination against orthopoxviruses at least 7 days prior to mpox diagnosis

**Fig. 1:**
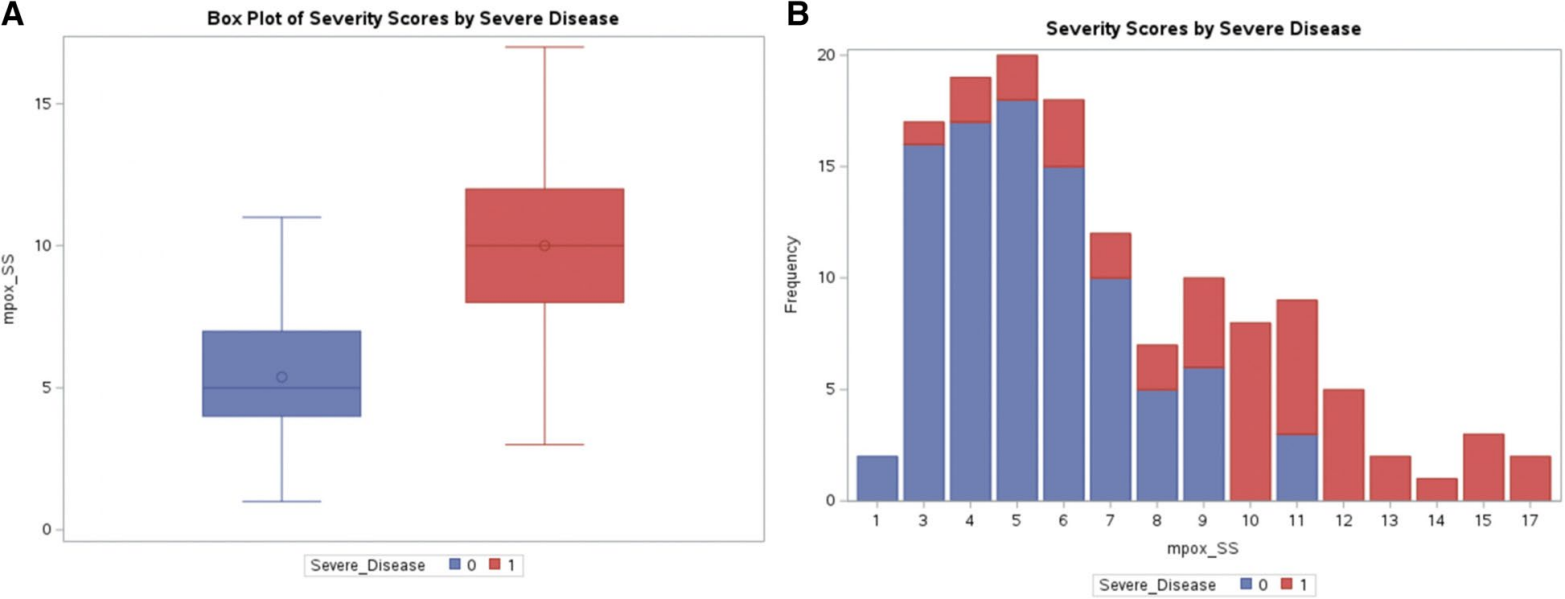
Mpox severity score distribution and correlation of mpox severity score with severe disease outcome variable. **A** The box plot shows those with severe mpox disease had higher mpox severity scores than those who did not develop severe disease. **B** This chart illustrates the distribution of the mpox severity score, overlayed by the composite severe disease variable. 0: not severe disease. 1: severe disease

For the 135 patients included in the mpox-SS analysis, the overall mean (SD) mpox-SS was 6.85 (3.36). The mean (SD) mpox-SS was 5.38 (2.1) for non-severe and 10.0 (3.39) for severe (*p* < 0.0001, Fig. 1), as defined by composite severe mpox disease. Additionally, Fig. 1 shows stacked bar charts of mpox-SS by our composite severe disease variable with a cut-off around 8 between severe and non-severe disease.

### Regression analysis

In univariate analysis, insurance status was significantly associated with severe mpox disease (Table 3). The model had an overall *F* value of 5.6 and an *R*-squared value of 0.04. The addition of high-risk condition, presence of STI, prior JYNNEOS vaccination, and race/ethnicity (Black and Hispanic) increased the *R*-squared to 0.12, and high-risk condition (*p* = 0.03) and prior vaccination (*p* = 0.02) were significant. Insurance status remained significant in the model after adjustment (*p* = 0.03).

**Table 3:** Univariable and multivariable linear regression analysis for association between insurance and mpox severity score (*N* = 135)

Variable	Unadjusted model^f^		Adjusted model^f^	
	*β* coefficient (95% CI)	*p* value	*β* coefficient (95% CI)	*p* value
Insurance^a^	− 1.51 (− 2.77, − 0.25)	0.02	− 1.42 (− 2.70, − 0.14)	0.03
High risk^b^			1.47 (0.12, 2.82)	0.03
STI^c^			− 0.11 (− 1.39, 1.81)	0.87
Prior vaccination^d^			− 1.73 (− 3.22, − 0.23)	0.02
Hispanic			0.21 (− 1.13, 1.56)	0.75
Black			− 0.87 (− 2.45, 0.70)	0.28
SVI^e^			1.79 (− 1.73, 5.31)	0.32

Referent categories: insurance = uninsured/Medicaid/AIDS Drug Assistance Program; high risk = no; STI = no; prior vaccination = no; Hispanic = White, Black = White

^a^Insurance represents private or Medicare; the referent is uninsured, Medicaid, and ADAP

^b^HIV and either CD4 < 350 cells/mm^3^ OR active AIDS-defining illness including mycobacterium avium complex, cryptococcal disease, Kaposi’s sarcoma, endemic fungal disease; solid organ or hematological transplantation; high dose steroids; skin-compromising condition

^c^STI (sexually transmitted infection) includes chlamydia, gonorrhea, syphilis, herpes simplex virus, or human papillomavirus

^d^Prior vaccination was coded as yes if the patient had at least one documented vaccination against orthopoxviruses at least 7 days prior to mpox diagnosis

^e^SVI = Social Vulnerability Index (continuous variable)

^f^Models adjusted for all listed covariates. Unadjusted model R-square 0.04; adjusted model R-square = 0.12, F = 0.02 p<0.05

## Discussion

This study is one of the few to explore associations between non-clinical factors beyond race/ethnicity with severe mpox disease. Our analysis showed that lack of insurance or having Medicaid was significantly associated with severe mpox outcomes, controlling for race/ethnicity, high-risk condition (inclusive of advanced HIV), STI, and prior vaccination. Our findings are not consistent with Garneau et al., which did not find an association between insurance status and hospitalization in an urban cohort [35]. Their analysis, however, did find an association between hospitalization and immunosuppression, consistent with our finding that high-risk condition is significantly associated with disease severity. To our knowledge, there is not a consistent variable for “severe mpox disease” in the literature, so our novel use of a composite variable considering both hospitalization and tecovirimat use is meant to add to the general discourse regarding how to operationalize severity. Furthermore, our study used a validated mpox-SS which considers the burden of skin lesions, confluent lesions, treatment for bacterial superinfection, mucosal areas affected, and analgesia requirement, in addition to hospitalization. Thus, it is possible the mpox-SS includes more granularity when it comes to physical markers of disease (e.g., skin lesions) rather than hospitalization. Multiple studies have begun to utilize the mpox-SS as a more objective marker of disease severity [20–22].

Lack of insurance or Medicaid status may have led to delayed access to care or delayed care seeking behavior during initial symptoms. Given that many clinicians and health systems were unprepared for the extent of the mpox outbreak or had challenges accessing tecovirimat, this may have left people without private insurance with heightened vulnerability to more severe disease. Our findings suggest that during an outbreak like mpox, understanding the role that insurance status plays in predicting disparate clinical outcomes is vital for policies that support an equitable response.

We acknowledge that NYC, the key epicenter of mpox cases, is a unique setting, and careful consideration is needed before generalizing our findings to other settings. NYC’s early access to testing, medications such as tecovirimat, availability of safety-net services, and tailored outreach to high-risk communities may have contributed to clinical outcomes in ways different from other regions with a delayed or uncoordinated response.

### Limitations

This study has limitations. The small sample size of our cohort limited the power of our statistical analysis. Second, there was a high number of observations with unknown status for some variables in our chart review due to a lack of consistent recording of sociodemographic variables. Note that these observations, documented as variables with “unknown” status, as mentioned in our study methods, were not truly missing but rather not documented in the electronic medical record (EMR). Manual chart review was unable to clarify sex at birth and gender identity for about 10% of our sample, leaving status for these variables unknown; however, available data about the overall mpox outbreak in the USA is consistent with > 90% cisgender men and > 90% male sex at birth. In addition, sociodemographic variables may be misclassified within the chart as these variables may not be frequently updated. Mpox-SS was calculated at different time points for each patient given that the study was retrospective and was not monitored serially over time. We were not able to calculate mpox-SS for 8 patients in the cohort due to limited clinical information. Additionally, age is a potential modifier given that people over age 50 likely were inoculated with the smallpox vaccine as children, theoretically providing some protection against mpox [36]. However, the number of participants in our cohort over age 50 was too small for analysis. Another limitation is that individual patient charts were only reviewed by one author; however, we implemented measures such as creating a data entry codebook and reviewing unclear patient charts as a group to ensure the accuracy of data extraction.

Lastly, the way we chose to operationalize and dichotomize insurance status may have influenced the results in our model.

## Conclusions

In summary, this study suggests being uninsured or on Medicaid is associated with worse mpox-related outcomes compared to privately insured or Medicare patients. To build on these findings, future studies should examine data from multiple hospital systems to understand the role of insurance as a predictor of severe disease and possibly consider other socioeconomic factors such as immigration status or preferred language. Additional attention is warranted to develop equitable policies to ensure protection of patients with this structural vulnerability.

## Data Availability

No datasets were generated or analysed during the current study.

## Abbreviations

ADAP: AIDS Drug Assistance Program
EA-IND: Expanded access investigational new drug
EMR: Electronic medical record
ESL: English as a second language
IPC: Infection prevention and control
IRB: Institutional Review Board
Mpox-SS: Mpox severity score
SMM: Sexual minority men
NYC: New York City
PCR: Polymerase chain reaction
PHL: Public health laboratory
SD: Standard deviation
